# Social Impact of Leishmaniasis, Afghanistan

**DOI:** 10.3201/eid1104.040945

**Published:** 2005-04

**Authors:** Richard Reithinger, Khoksar Aadil, Jan Kolaczinski, Mohammad Mohsen, Samad Hami

**Affiliations:** * HealthNet International, Peshawar, Pakistan

**Keywords:** health education, leishmaniasis, prevention, Leishmania tropica, Afghanistan, stigma, control, letter

**To the Editor:** For almost a decade, Kabul, Afghanistan, has had the highest incidence of cutaneous leishmaniasis in the world, with an estimated 67,500 to 200,000 cases each year (*1**–**3*). Because of sandfly vector exposure, most leishmaniasis lesions occur on the face; anecdotal reports of severe stigma are associated with the disease (*3*). To prioritize aspects of operational activities and before developing a disease-specific health education strategy, we collected data on knowledge, attitudes, and perceptions regarding leishmaniasis.

In October 2002, we randomly chose 5 of Kabul's 14 administrative districts to carry out a house-to-house survey (HHS) as well as 13 focus group discussions (FGDs) with women. The 5 districts chosen were Karti-Seh (HHS) and Dasht-e-Barchi (4 FGDs), Karti-Now (3 FGDs), Arzam Qemat (3 FGDs), and Rahman Mena (3 FGDs). The survey was conducted by using a standardized, multiple-choice questionnaire. The most senior, available family member in 252 neighboring households was interviewed, after the first household was randomly selected (*2*). We focused on women in FGDs because they have greater risk for leishmaniasis than men (*2**,**3*) and are often the primary caregivers in Afghan culture (*4*). The same HHS questions were used in the FGDs. Surveyors randomly chose a house in each district and explained the study's purpose to residents. When residents agreed to host an FGD, women from neighboring households were invited to join. FGDs had a maximum of 12 participants and lasted 2 hours; answers to questions were recorded on paper. FGD moderators were instructed to pose questions, encourage free discussion, and ask participants to emphasize personal experiences. FGD data were analyzed by thematic analysis of the transcripts. Surveys were carried out by experienced surveyors, who have been involved in previous leishmaniasis prevalence surveys or intervention trials (*2**–**4*). Written approval for the study was obtained from the Afghan Ministry of Health, and oral consent was given by all surveyed persons. Active case-patients surveyed were offered free antileishmanial treatment at the HealthNet International leishmaniasis clinics.

A total of 252 and 108 persons were surveyed in the HHS and FGDs, respectively, although not all respondents answered every question. Our study confirmed the prevalence of cutaneous leishmaniasis in Kabul; 128 (51%) of 252 HHS respondents reported a family member with leishmaniasis. Respondents were knowledgeable about leishmaniasis: of 360 total HHS and FGD respondents, 287 (80%) said that it was a disease, and 160 (44%) said that it was acne. Of 66 FGD respondents who knew that leishmaniasis was a disease, 29 (44%) knew that it was transmitted by mosquitoes. Of 104 FGD respondents, 41 (43%) could describe the clinical symptoms of leishmaniasis (each was asked to give 1 answer only), i.e., an open wound (n = 17) that is not painful (n = 7) and takes a long time to cure (n = 17).

The principal finding of our study is that we show, for the first time, the extent of the disease's social impact in Kabul. Because erroneous beliefs exist that the disease can be transmitted by person-to-person physical contact (of 360 respondents, the most common answers were "touching" [n = 86] and "sharing meals and household goods" [n = 26]), affected people are excluded from communal life. This exclusion can consist of minor domestic restrictions (40 [46%] of 89 FGD respondents said they would not share plates, cups, or towels with leishmaniasis patients) or more severe measures that lead to physical and emotional isolation. FGDs showed that leishmaniasis caused trauma; of 83 respondents who had children with leishmaniasis, 45 (54%) said their children felt disfigured because of lesions or scars (n = 20), because of painful treatment (intralesional or intramuscular injections with pentavalent antimony, n = 19), or because they were excluded from play with other children (n = 6). Of 96 FGD respondents, 21 (22%) said that a mother with leishmaniasis should not breast-feed her child; 48 (51%) of 94 FGD respondents would prevent someone with leishmaniasis from touching or hugging their children; 55 (57%) of 96 respondents said that a person with leishmaniasis should not be allowed to cook for the family; and 21 (22%) of 94 respondents said that a woman with a leishmaniasis lesion or scar will have difficulty finding a husband. Severity and visibility of the lesions as well as past experience of leishmaniasis within the family influenced respondents' answers.

The study yielded 2 other important findings. First, 245 (97%) of 252 HHS respondents knew that leishmaniasis does not resolve without treatment and that patients should seek professional assistance. Of 344 HHS and FGD respondents, 322 (94%) said that leishmaniasis patients should seek a doctor or clinic for treatment (as opposed to a traditional healer or self-medication). Second, 205 (57%) of 358 HHS and FGD respondents use methods to prevent exposure to sandfly vectors, i.e., screens for windows and doors (n = 108), nets around beds (n = 63), indoor insecticide spraying (n = 24), or other method of personal protection (n = 10); 152 (78%) of 252 HHS respondents said that they did not have a net over their bed because it was too expensive.

Kabul residents are knowledgeable about leishmaniasis; they are able to describe its symptoms and the necessity for professional treatment. However, we show that while many FGD respondents knew that leishmaniasis is transmitted by "mosquitoes," severe stigma and trauma are associated with the disease, particularly in children and women. Our operational experience corroborates this finding, which underlines the disease's social effect on the local population and refuting the belief that leishmaniasis is of little health importance (*5*). Half of the 15,983 leishmaniasis patients treated at HNI clinics in 2003 were women. Although women are at greater risk for leishmaniasis, they do not typically attend healthcare programs in Afghanistan because of sociocultural constraints (e.g., husbands not allowing their wife or daughters to attend) (*6*). In addition to diagnosing and treating active cases, HealthNet International will now focus on leishmaniasis education activities in Kabul, outlining aspects of disease transmission and prevention, as well as disseminating messages to reduce the disease's social impact.
